# Comparison of gender, age, and body mass index for spatiotemporal parameters of bilateral gait pattern

**DOI:** 10.12688/f1000research.51700.2

**Published:** 2022-12-12

**Authors:** Turki Abualait, Mohammad Ahsan

**Affiliations:** 1 Department of Physical Therapy, College of Applied Medical Sciences, Imam Abdulrahman Bin Faisal University, Dammam, Saudi Arabia

**Keywords:** Spatiotemporal Parameters, Gait, GaitUp, Gender, Age, Body Mass Index

## Abstract

**Background:** Studies on the gaits parameters have been identified on the patient population. Most researchers confirm that the patients walk differently than normal people and they may have a greater risk of falls. Consistent finding and description of gender, age, and body mass index differences in gait studies is rare in healthy subjects.

This research was performed to compare spatiotemporal parameters of gait between gender, as per their age and body mass index level.

**Methods: **A cross-sectional study was conducted with forty-five young adults (F=20, M=25). Stadiometer and Physilog 4 inertial sensors were used for data collection. A gait analyzer 5.2 software (GaitUp, S.A. Lausanne, Switzerland) was used to determine spatiotemporal parameters.

**Results: ** Females were found with higher mean score of total double support and cadence than males. Cadence also increases with age. Obese people showed lower gait speed, cadence, and total double support. No statistically significant differences were found in any bilateral foot gait parameters with respect to gender, age, and body mass index.

**Conclusion:** The findings of the study may be beneficial to those who have abnormal gait pattern due to age, body mass index differences, decreased muscle strength, spasticity, and joint mobility. This important information should be considered to rehabilitate patients with abnormal gait patterns to controlling dynamic balance and risk of falling.

## Introduction

Spatiotemporal is the primary measurement in gait analysis. There is a common perception that males walk differently from females. The differences may occur due to physical capacities such as muscular strength, endurance, coordination, flexibility, agility, and emotional balance. Walking patterns may also fluctuate by age, body mass index (BMI), surface, course of time, and changes from stride to stride. Gender differences in a healthy population reveal contradictory discoveries regarding spatiotemporal parameters of gait. ^1^

Many studies have either been surprisingly limited in their investigation, contradictory, or equivocal between genders. One study reported that stride characteristics do not have any gender differences during walking. ^2^ Another study revealed that during walking, the speed is the same in males and females, but that the step length is shorter in females. ^3^ On the other hand, a study reported males to walk faster than females, and that female step length is shorter than males. ^4^ A previous study concluded that healthy older females walk with shorter stride length and higher cadence when compared to males. ^5^ Females have been indicated to walk with lower preferred speed, smaller step length, but increased cadence compared with males. ^6^ It has also been reported that healthy females had a higher velocity and bigger cadence, swing phase, stride length, and single support phase and a lower double support phase and stance phase compared with gait disorder. ^7^ Heredia-Jimenez and Orantes-Gonzalez reported that while dealing with healthy females and females with fibromyalgia, there were significant differences in stride length, velocity, swing time variability, cadence, and stance gait. ^8^

Advance aging factors affect gait pattern. Many studies have been reported that gait ability declines with age. A prior study found that age differences are connected with slower gait speed, shorter stride length, and wider stride width. ^9^ Compared to young adults, the elderly adult gait model is characterized by shorter step length, slow gait speed, and a reduced range of motion at the hip. ^10^ A study showed that when walking on a compliant surface, young and older people increase cadence and reduce velocity. ^11^ One of the most consistent age-related changes has been shown as a decline in gait speed. ^12^ There are many fall injuries associated with increasing age, from young adults to middle-aged adults to older adults, as the aging process is accompanied by changes in body composition. ^13^

Obesity is a primary risk factor for many diseases which also negatively affects physical functioning, especially walking ability and performance. ^14^ The effects of obesity and overweight on gait parameters in adults not well known. In obese adults, gait is distinguished by slow step frequency, shorter step length, longer stance phase, and walking speed is minimized. ^13^ Stride frequency and stride length did not differ between moderately obese individuals and healthy weight individuals. ^15^ Obese individuals walk slowly with a shorter step length than underweight individuals. ^16^ There is a lack of knowledge on spatiotemporal parameters from the underweight, healthy, overweight, and obese population.

Many methods were suggested to investigate the spatiotemporal patterns of the gait sequence to understand the differences between males and females. Thang et al. used only the smartphone's accelerometer sensor to user authentication while Zong and Deng captured walking information from both the accelerometer and gyroscope sensors. ^17^^,^^18^ Recently, the Physiolog gait analysis system from GaitUp (SA, Lausanne, Switzerland) has become a popular and is an important tool for the objective evaluation and planning of rehabilitation strategies for an abnormal gait pattern. The use of these sensors has been described previously, and they demonstrate good accuracy and perception of gait analysis. ^19^^,^^20^ Despite this evidence, a limited number of studies have investigated the spatiotemporal parameters of gait using internal wearable sensors.

Therefore, the purpose of this current study was to compare the characteristics of spatiotemporal gait based on gender, age, and BMI level. In particular, the aim of the study is to answer the question of whether there are differences between gait parameters. To achieve the objective of this study for gait parameters in gender, age, and BMI level, we tried a statistical comparison between males and females, age differences, and BMI categories for spatiotemporal gait analysis.

## Methods

### Study design

A cross-sectional study design was chosen to achieve the objective of this study. This study was conducted in accordance with the Declaration of the Principles of Helsinki.

### Ethical statement

This study was approved by the local Institutional Review Board at Imam Abdulrahman Bin Faisal University with IRB-Number: IRB-2019-03-255.

### Participants

45 young adults (25 male and 20 female) were included in this study. Participants were recruited through an open advertisement within the university campus. Their mean (standard deviation [SD]) age, height, weight, and BMI were 21.82(3.93) years, 165.83(8.00) cm, 66.10(13.19) kg, and 24(3.89) respectively. The study was carried out from June to August of 2019, data were collected during single session for each participant at biomechanics gait lab in Imam Abdulrahman Bin Faisal University. All participants who met the inclusion criteria had no history of musculoskeletal or neurological deficits which could affect their gait performance, and all were able to understand and follow commands. Exclusion criteria were any significant gait-associated impairments or any previous injury that has an effect on gait performance, psychiatric illness and severe cognitive deficits. All participants wear flat shoes during taking the assessment to standardize the procedure of the test and minimize the effects of any footwear on gait performance. No bias were identified that would affect this study.

**Table 1.** Anthropometric measurement of participants.

	Male (N=25) Mean ± SD	Female (N=20) Mean ± SD	Both (N=45) Mean ± SD	Sig.
**Age (Years)**	24.88 ± 5.60	23.75 ± 6.41	24.72 ± 6.43	.314
**Height (cm)**	170.78 ± 6.25	159.65 ± 5.15	165.83 ± 8.00	.000
**Weight (kg)**	71.90 ± 12.40	58.85 ± 10.45	66.10 ± 13.19	.001
**Leg length (cm)**	99.7 ± 2.6	91.6 ± 2.7	95.65 ± 2.65	.084
**BMI**	25.53 ± 3.92	22.80 ± 3.37	24.32 ± 3.89	.018

Table 1 showed significant differences in height (p = .000) and weight (p = .001) for male and female participants. Age, leg length, and BMI level showed insignificant differences for both genders.

## Equipment

### Stadiometer cum weighing scale

Weight, height, and BMI were measured with a portable electronic calibrated stadiometer cum weighing scale (Detecto Scale-model 750, USA). Participants were asked to wear light clothing and take off their shoes for accurate measurement.

### Physiolog 4

To measure spatiotemporal parameters of all participants Physilog 4 silver 10D from GaitUp (S.A., Lausanne, Switzerland) was used. Physilog has good accuracy and precision for gait analyses. ^19^^,^^20^ Physilog is the high-quality 3D accelerometer, 3D gyroscope and barometric pressure sensor. It provided raw 3D acceleration data at a sample rate of 128 Hz. It has inbuilt SD memory card to store recorded data. Gait analyser 5.2 application for gait was installed on a tablet/computer, and it was connected with sensors. Stored data were transfer to the computer through the USB cable. The Physilog form GaitUp gait analysis is a valid and reliable tool to measure spatiotemporal parameters. ^21^^,^^22^

### Procedure

A total of 45 participants agreed to participate in the study. There were 20 female and 25 male participants. Before the actual test, all basic instructions were explained to the participants. After collecting their anthropometric data with a stadiometer cum weighing scale, physilog inertial sensors were placed on each participant's foot for the gait test. Participants were asked to walk at a comfortable pace for themselves, along a 10-meter straight path. The three trails were recorded. The average of the three trials was used for further analysis. In this study, the spatiotemporal parameters chosen for the analysis were gait speed, gait cycle, foot speed (left & Right), stride length, total double support, and cadence for both legs. A gait analyser 5.2 software (GaitUp, S.A. Lausanne, Switzerland) was used to determine Gait Speed (meter/second) is the product of distance cover by one foot in one second, Gait Cycle (seconds) The gait cycle is the time span between the stance phase and the swing phase of the same foot, Foot Speed (meter/second) the maximum speed during the swing phase, and stride length (meter) the distance covered by one foot, Total Double Support (% Cycle) when the both feet on the ground, and Cadence (Step/Min) steps per minutes. ^23^

## Statistical analysis

Statistical analysis was performed using IBM SPSS for windows, version-21 (IBM Crop. USA). Prior to analysis, data were screened for missing values and outliers. Descriptive analyses were conducted for anthropometric characteristics. Independent sample t-tests and one-way analysis of the variance (ANOVA) test was extended to find out the differences between different types of spatiotemporal parameters of gait for gender, age, and BMI levels. The significance level was set at the 0.05 level.

## Results

**Table 2.** A comparative analysis between male and female participants.

Gait Parameters	Foot type	Males (N=25) Mean ± SD	Females (N=20) Mean ± SD	Std. Error Difference	Sig.
Gait Speed (M/S)	Left	1.18 ± 0.10	1.16 ± 0.12	0.033	0.631
	Right	1.17 ± 0.12	1.13 ± 0.12	0.036	0.360
Gait Cycle (Sec.)	Left	1.14 ± 0.11	1.12 ± 0.08	0.029	0.358
	Right	1.15 ± 0.10	1.15 ± 0.10	0.030	0.926
Foot Speed (M/S)	Left	3.71 ± 0.37	3.62 ± 0.32	0.103	0.373
	Right	3.64 ± 0.49	3.62 ± 0.32	0.126	0.904
Stride Length (M)	Left	1.29 ± 0.09	1.28 ± 0.06	0.024	0.764
	Right	1.29 ± 0.09	1.25 ± 0.06	0.025	0.176
Total Double (%Cycle)	Left	20.02 ± 3.81	21.67 ± 3.65	1.121	0.147
	Right	19.99 ± 3.81	21.67 ± 3.65	1.122	0.140
Cadence (Step/Min)	Left	108.13 ± 6.85	108.60 ± 7.97	2.210	0.834
	Right	108.83 ± 6.07	108.88 ± 7.66	2.045	0.981

The results of the gait parameters for males and females' scores are presented in Table 2. When considering the mean score, males have higher gait speed, gait cycle, foot speed, and stride length than females, while females have higher total double support and cadence than males. There were no significant differences found in any gait parameters in the left and right foot for male and female participants.

**Table 3.** A comparative analysis across age categories.

Gait Parameters	Foot type	Age (18-25) (N=31) Mean ± SD	Age (30-38) (N=14) Mean ± SD	Std. Error Difference	Sig.
Gait Speed (M/S)	Left	1.18 ±0.12	1.15 ± 0.07	0.036	.413
	Right	1.15 ± 0.14	1.15 ± 0.08	0.039	.831
Gait Cycle (Sec.)	Left	1.13. ± 0.10	1.13 ± 0.08	0.031	.918
	Right	1.14 ± 0.11	1.15 ± 0.09	0.032	.849
Foot Speed (M/S)	Left	3.70 ± 0.38	3.61 ± 0.24	0.111	.454
	Right	3.68 ± 0.38	3.51 ± 0.48	0.132	.203
Stride Length (M)	Left	1.30. ± 0.09	1.26. ± 0.04	0.025	.182
	Right	1.28. ± 0.10	1.26. ± 0.04	0.027	.450
Total Double Support (%Cycle)	Left	20.93 ± 3.65	20.37 ± 4.19	1.230	.652
	Right	20.90 ± 3.66	20.37 ± 4.19	1.233	.666
Cadence (Step/Min)	Left	108.27 ± 7.97	108.48 ± 5.76	2.373	.931
	Right	108.77 ± 7.42	109.03 ± 6.74	2.194	.906

The result of gait parameters based on age categories (18-25 and 30-38 years) scores are presented in Table 3. When considering the mean scores, the participants belonging to the 18-25 age category showed higher gait speed, gait cycle, foot speed, stride length, and total double support than participants belonging to the 30-38 age category. While the participants belonging to the 30-38 age categories showed higher left and right foot’s cadence than the participants belong to the 18-25 age category. The results showed that there were no significant differences found among any gait parameters in left and right foot for either age category.

The result of gait parameters based on their BMI levels (underweight, healthy, overweight, and obese) scores are presented in Table 4. When considering the mean scores, the participants according to their BMI level showed that underweight participants have the highest gait speed, foot speed, stride length, and cadence than the participants belong to other BMI categories. While the gait cycle and total double support for the left and the right foot are smaller than the other BMI categories participants. The participants belonging to the healthy BMI category show that the gait speed, foot speed, stride length, and cadence are higher, and their gait cycle is smaller than overweight and obese participants, while total double support for the left and the right foot is lower than overweight and higher than obese participants. The overweight participants showed the gait speed, gait cycle, foot speed, and the stride length are lower than obese participants, while total double support and cadence for left and right were higher for overweight participants than obese participants. The obese participants showed gait speed and cadence are lower and the gait cycle is higher than other BMI categories participants, foot speed is higher than healthy and overweight participants while lower than underweight participants. The total double support was lower than healthy and overweight participants but higher than underweight participants. The results showed that there were no significant differences in any gait parameters for the left and right foot for any BMI categories’ participants.

**Table 4.** A comparative analysis between body mass index (BMI) level.

Gait Parameters	Foot type	Underweight (N=2) Mean ± SD	Healthy (N=28) Mean ± SD	Overweight (N=11) Mean ± SD	Obese (N=4) Mean ± SD	Sig.
Gait Speed (M/S)	Left	1.26 ± 0.12	1.18 ± 0.10	1.15 ± 0.13	1.11 ± 0.11	.371
	Right	1.19 ± 0.08	1.17 ± 0.10	1.11 ± 0.16	1.13 ± 0.17	.558
Gait Cycle (Sec.)	Left	1.04 ± 0.06	1.13 ± 0.08	1.15 ± 0.12	1.17 ± 0.16	.471
	Right	1.14 ± 0.09	1.13 ± 0.09	1.17 ± 0.10	1.17 ± 0.17	.737
Foot Speed (M/S)	Left	3.84 ± 0.14	3.70 ± 0.35	3.52 ± 0.36	3.82 ± 0.24	.314
	Right	3.78 ± 0.33	3.62 ± 0.47	3.57 ± 0.36	3.78 ± 0.23	.821
Stride Length (M)	Left	1.31 ± 0.05	1.29 ± 0.09	1.26 ± 0.08	1.29 ± 0.06	.664
	Right	1.24 ± 0.03	1.29 ± 0.07	1.24 ± 0.11	1.26 ± 0.07	.444
Total Double Support (%Cycle)	Left	16.03 ± 0.17	20.82 ± 3.50	21.99 ± 3.53	19.24 ± 5.94	.178
	Right	16.03 ± 0.17	20.79 ± 3.15	21.99 ± 3.53	19.24 ± 5.94	.179
Cadence (Step/Min)	Left	115.56 ± 6.60	108.64 ± 6.08	107.17 ± 8.08	105.83 ± 12.86	.441
	Right	116.04 ± 5.56	109.10 ± 5.99	107.31 ± 7.28	107.80 ± 10.51	.405

## Discussion

The results suggest that there was no significant difference in male and female participants for the left and right foot gait parameters. The findings of this study indicated that gait speed, gait cycle, foot speed, and stride length were overall higher in male than female participants. Whereas cadence and double support were higher in female than male participants. Most previous studies suggested that step length and cadence are responsible for the gait speed, and these measurements have some biological dependence on the height of the individual. ^24^ Kerrigan *et al* have observed that healthy males who walked at the same walking speed as females showed lower cadence and longer step length than the female. ^25^ It was reported that the healthy weight category women walk with reduced stride length and higher cadence with respect to men, in order to achieve comparable speed values. ^5^ It was reported that the spatiotemporal gait parameters for both genders showed that females have greater stride time while males performed higher stride length, step time, cadence, and walking speed. ^26^ Kerrigan *et al* revealed that there are few significant gender differences for spatiotemporal data, with a longer normalized stride length and greater cadence in females. Both genders had the same step width and walking velocity due to the effort that females made to increase their stride length with the aim of walking as fast as males. ^25^ We found that males and females did not differ significantly in the spatiotemporal parameters of normal gait speed, gait cycle, normal stride length, and cadence. These findings are in partial agreement with the findings of the above-mentioned studies. Gender differences may also be associated with body proportions between males and females. Muscle strength and bone configuration may have importance to determine gait parameters outcomes between genders.

The results of gait parameters based on two age categories (18-25 and 30-38 years old) scores are presented in Table 3. The results of the present study indicated that there were no significant differences in between any spatiotemporal parameters in the age groups. The participants belonging to the 18-25 age category showed higher gait speed, gait cycle, foot speed, stride length, and total double support than participants belonging to the 30-38 age category. However, the participants belonging to the 30-38 age categories showed higher left and right foot’s cadence than the participants belonging to the 18-25 age category. Several studies indicated that these spatiotemporal measures deteriorate more rapidly with age for women than for men, ^27^ while others found no interactions between the sexes during aging. ^28^ Results from Frimenkoa *et al* study indicated that there was a significant difference in both genders which slowed their gait speed with age. At a similar age, females have a higher cadence and smaller stride lengths than males. As the age increases, gait speed decreases in both genders, while females maintain smaller step length and higher cadence. ^29^ Moreover, Abreu and colleagues uncovered a negative relationship between aging and stride length during gait due to increased eccentric activity of the quadriceps muscles during the final stage of double support or increased eccentric activity in the hamstrings during the final balance phase that occurs with increasing age. ^30^ Older adults reduce their gait speed and take shorter steps while increasing the time of double support to maintain their dynamic balance. ^31^ The results of the present study also support previous findings that age-related changes in gait speed through shorter steps were adopted for a safer and steadier gait.

Our findings showed that the underweight participants have the highest gait speed, foot speed, stride length, and cadence than the participants belong to other BMI categories, while gait cycle and total double support for the left and the right foot are smallest than the other BMI categories participants. The participants belonging to the healthy weight category showed that the gait speed, foot speed, stride length, and cadence are higher, and the gait cycle is smaller than the overweight and obese category participants. However, their total double support for the left and the right foot is lower than the overweight category participants and higher than the obese category participants. The overweight category participants demonstrated that the gait speed, gait cycle, foot speed, and the straight length is lower than the obese category participants, while total double support and cadence for left and right showed higher for the overweight category than the obese category participants. The obese category participants showed gait speed and cadence are lower and the gait cycle is higher than other BMI categories participants, foot speed is higher than the healthy weight and overweight participants while lower than the underweight participants. The total double support was lower than the healthy weight and overweight categories but higher than the underweight category participants.

People who are overweight and/or obese are known to have a functional implication in everyday life. It has been shown that excess weight alters the normal gait mechanism. ^16^ Our findings are in line with previous research, with obese adults walking with shorter strides in length, large stride width, and shorter stride length compared to healthy weight adults when walking at a self-defined speed. ^32^ On the other hand, when comparing healthy weight and obese adults, no differences were found in step length. ^16^^–^^17^ However, these results may be directly due to the effect of speed. ^16^ It has been clearly indicated in the literature that obese people tend to have reduced stride length, swing phase duration, cadence, walking speed, increased stance phase, step width, and double support. ^33^ A recent review summarizing the results of 25 studies on the gait of obese children ^34^ concluded that there is moderate evidence of increased step width and stance phase duration, while for all other spatiotemporal parameters, the differences are either non-significant or inconsistent as our results suggest.

### Limitations

Some limitations should be considered which might limit the generalizability of the findings. First, all participants were adults; different ages and weights categories were not involved in the study. Second, Physilog (GaitUp) is not a common device for determining spatiotemporal gait parameters, however this may be employed more in future research. Sample size was small in this research due to sample size was not calculated. Might be we can find significant differences if the sample size will be large. Finally, no comparable group with gait disturbances was enrolled in this study to compare the spatiotemporal gait parameters with healthy subjects. Thus, future studies should compare the spatiotemporal parameters of gait between gender, as per their age and body mass index level between healthy adults and patient groups. Future research should focus on more frail population to see if the spatiotemporal gait parameters differences existed among them and how these effect on their quality of life.

## Conclusions

In summary, we quantified the spatiotemporal parameters of gait differences as per gender, age, and BMI levels. Our result suggests that there were no statistically significant differences in all the spatiotemporal parameters for gender, age, and BMI levels at the left and right foot. These findings may be beneficial to those who have abnormal gait pattern due to age, BMI differences, decreased muscle strength, spasticity, and joint mobility. This important information should be considered when rehabilitating patients with abnormal gait patterns with to controlling dynamic balance and risks of falling.

## Data availability

### Underlying data

Harvard Dataverse: “Comparison of Gender, Age, and Body Mass Index for Spatiotemporal Parameters of Bilateral Gait Pattern”, https://doi.org/10.7910/DVN/10WUSP. ^36^

This project contains the following underlying data:

• Raw data excel file

Data are available under the terms of the Creative Commons Zero “No rights reserved” data waiver (CC0 1.0 Public domain dedication).

## Consent statement

Participants who agreed to participate in the study voluntarily were given a detailed written and verbal explanation of the study then asked to sign a written consent form.
