# Supplementary material for: Comparison of gender, age, and body mass index for spatiotemporal parameters of bilateral gait pattern
Source: F1000Res. 2022 Dec 12;10:266. Originally published 2021 Apr 1. [Version 2] doi: 10.12688/f1000research.51700.2 (PMC9623194; doi:10.12688/f1000research.51700.2)
Supplement: Peer review history

# Peer review: Reviewer response for version 2

**DOI:** 10.5256/f1000research.141855.r157986

**Published:** 2023-01-10

**Authors:** Hajra Masood

**Affiliations:** 1 Department of Computer Science, Bahria University Karachi, Karachi, Pakistan

The revised version of research paper is compete and comprehensive. I recommend acceptance of the paper in current form.

Is the work clearly and accurately presented and does it cite the current literature?

Yes

If applicable, is the statistical analysis and its interpretation appropriate?

Yes

Are all the source data underlying the results available to ensure full reproducibility?

Yes

Is the study design appropriate and is the work technically sound?

Yes

Are the conclusions drawn adequately supported by the results?

Yes

Are sufficient details of methods and analysis provided to allow replication by others?

Partly

Reviewer Expertise:

computer vision , machine learning , gait analysis.

I confirm that I have read this submission and believe that I have an appropriate level of expertise to confirm that it is of an acceptable scientific standard.

# Peer review: Reviewer response for version 2

**DOI:** 10.5256/f1000research.141855.r157985

**Published:** 2022-12-19

**Authors:** Haruhiko Sato

**Affiliations:** 1 Faculty of Rehabilitation, Kansai Medical University, Hirakata, Japan

At the beginning of the results section, the authors stated "When considering the mean score, males have higher gait speed, gait cycle, foot speed, and stride length than females, while females have higher total double support and cadence than males”, but the values (the mean values and the p values) were not provided. Did the differences reach statistically significant? Again, the authors should discuss the differences based on the statistical significance.

Is the work clearly and accurately presented and does it cite the current literature?

Partly

If applicable, is the statistical analysis and its interpretation appropriate?

Partly

Are all the source data underlying the results available to ensure full reproducibility?

Partly

Is the study design appropriate and is the work technically sound?

Partly

Are the conclusions drawn adequately supported by the results?

No

Are sufficient details of methods and analysis provided to allow replication by others?

Partly

Reviewer Expertise:

Physiotherapy

I confirm that I have read this submission and believe that I have an appropriate level of expertise to confirm that it is of an acceptable scientific standard, however I have significant reservations, as outlined above.

# Peer review: Reviewer response for version 1

**DOI:** 10.5256/f1000research.54892.r153602

**Published:** 2022-11-08

**Authors:** Hajra Masood

**Affiliations:** 1 Department of Computer Science, Bahria University Karachi, Karachi, Pakistan

The study describes the effects of gender, age and obesity on spatiotemporal parameters of gait.

 The findings are backed with data and statistical analysis, but they lack logical discussion and a future direction for the research. The data and procedure of experiment is clearly mentioned but reproduction and verification of results is difficult as data collection is conducted on specialized equipment.

 The inferences made after research are generalized in nature.

Is the work clearly and accurately presented and does it cite the current literature?

Yes

If applicable, is the statistical analysis and its interpretation appropriate?

Yes

Are all the source data underlying the results available to ensure full reproducibility?

Yes

Is the study design appropriate and is the work technically sound?

Yes

Are the conclusions drawn adequately supported by the results?

Yes

Are sufficient details of methods and analysis provided to allow replication by others?

Partly

Reviewer Expertise:

computer vision , machine learning , gait analysis.

I confirm that I have read this submission and believe that I have an appropriate level of expertise to confirm that it is of an acceptable scientific standard, however I have significant reservations, as outlined above.

# Response

**Published:** 2022-12-05

**Authors:** Mohammad Ahsan

**Affiliations:** Imam Abdulrahman Bin Faisal University , Saudi Arabia

The study describes the effects of gender, age and obesity on spatiotemporal parameters of gait.

 The findings are backed with data and statistical analysis, but they lack logical discussion and a future direction for the research. The data and procedure of experiment is clearly mentioned but reproduction and verification of results is difficult as data collection is conducted on specialized equipment.

 The inferences made after research are generalized.

 Most of the changes have been made.

# Peer review: Reviewer response for version 1

**DOI:** 10.5256/f1000research.54892.r153604

**Published:** 2022-10-31

**Authors:** Hanatsu Nagano

**Affiliations:** 1 Institute for Health and Sport (IHES), Victoria University, Melbourne, VIC, Australia

Gait characteristics contain various health information and spatio-temporal parameters have been recognised as the fundamental descriptions of individuals’ walking patterns. The current research investigated a total of 45 healthy young adults’ spatio-temporal parameters sub-classified by gender, age and body mass index. While the study was the important topic, there are some fundamental concerns to be clarified.

 First, the study seemed to investigate effects of (i) gender, (ii) age and (iii) body mass index on spatio-temporal gait parameters. However, the total sample is only 45 and there seemed to be only the healthy young population included in the study. This limited sample may not be sufficient in leading any meaningful conclusion about age/body mass index effects on spatio-temporal gait parameters. There are the previous studies that have incorporated much larger samples about spatio-temporal parameters; therefore, the current study is expected to demonstrate the unique findings.

 Second, the introduction can be improved by elaborating on the three separate discussions about (i) gender, (ii) age and (iii) BMI effects on gait.

 Third, I wonder if there were any inclusion criteria in the study in terms of age. Were participants recruited widely from the community (e.g., targeting participation of 65+ yrs population)? The sample population seemed to be limited to the healthy and young.

 Fourth, some gait parameters were not defined properly. For example, what was foot speed? Was it the maximum speed during the swing phase, the average or something else? Similarly, what was Gait Cycle?

 Fifth, tables 2 & 3 should show sample sizes based on the subclassifications (i.e., age, BMI).

 Sixth, what was the importance of this study if no significant differences were identified in any way? Step length or other parameters may possibly reveal gender differences if normalisation techniques were employed.

Is the work clearly and accurately presented and does it cite the current literature?

No

If applicable, is the statistical analysis and its interpretation appropriate?

Yes

Are all the source data underlying the results available to ensure full reproducibility?

Yes

Is the study design appropriate and is the work technically sound?

Partly

Are the conclusions drawn adequately supported by the results?

Yes

Are sufficient details of methods and analysis provided to allow replication by others?

No

Reviewer Expertise:

Gait biomechanics, falls prevention, ergonomics, robotics, rehabilitation

I confirm that I have read this submission and believe that I have an appropriate level of expertise to confirm that it is of an acceptable scientific standard, however I have significant reservations, as outlined above.

# Response

**Published:** 2022-12-05

**Authors:** Mohammad Ahsan

**Affiliations:** Imam Abdulrahman Bin Faisal University , Saudi Arabia

Gait characteristics contain various health information and spatio-temporal parameters have been recognised as the fundamental descriptions of individuals’ walking patterns. The current research investigated a total of 45 healthy young adults’ spatio-temporal parameters sub-classified by gender, age and body mass index. While the study was the important topic, there are some fundamental concerns to be clarified.

 First, the study seemed to investigate effects of (i) gender, (ii) age and (iii) body mass index on spatiotemporal gait parameters. However, the total sample is only 45 and there seemed to be only the healthy young population included in the study. This limited sample may not be sufficient in leading any meaningful conclusion about age/body mass index effects on spatiotemporal gait parameters. There are the previous studies that have incorporated much larger samples about spatiotemporal parameters; therefore, the current study is expected to demonstrate the unique findings.

 Second, the introduction can be improved by elaborating on the three separate discussions about (i) gender, (ii) age and (iii) BMI effects on gait.

 Paragraph # 2 elaborates the gender and gait; Paragraph # 3 is related to age differences in gait parameters; paragraph # 3 is on BMI or obesity's effect on gait.

 Third, I wonder if there were any inclusion criteria in the study in terms of age. Were participants recruited widely from the community (e.g., targeting participation of 65+ yrs population)? The sample population seemed to be limited to the healthy and young.

 To recruit the participant an open advertisement was done within the university campus. We got participants only age range 19-38 only. 

 Fourth, some gait parameters were not defined properly. For example, what was foot speed? Was it the maximum speed during the swing phase, the average or something else? Similarly, what was Gait Cycle?

 Meaning defined added

 Fifth, tables 2 & 3 should show sample sizes based on the subclassifications (i.e., age, BMI).

 Added

 Sixth, what was the importance of this study if no significant differences were identified in any way? Step length or other parameters may possibly reveal gender differences if normalisation techniques were employed.

# Peer review: Reviewer response for version 1

**DOI:** 10.5256/f1000research.54892.r151494

**Published:** 2022-10-11

**Authors:** Haruhiko Sato

**Affiliations:** 1 Faculty of Rehabilitation, Kansai Medical University, Hirakata, Japan

I read with interest this research article that addressed the comparison of gender, age, and body mass index on spatiotemporal parameters of gait.

 My main concern is that the authors stated the conclusion not standing on their statistical results. In the statistical results of this study, there was no difference between gender to spatiotemporal parameters of gait, however, the authors seem to conclude gender differences exist as like the previous related articles.

 The following provides more specific comments by section:

**Abstract**

 Results: The authors stated, "Females were found with higher total double support and cadence than males." but this is not supported by the statistically significant result.

 Conclusion: It's unclear what refers to "these findings". It refers to "no differences for gender”? or refers to "females showed higher cadence than males"? The authors should state the conclusion more clearly and it should be based on the statistical results.

**Introduction**

 In the first paragraph, line 5: I think the authors should distinguish spatiotemporal parameters of gait (i.e., stride length or cycle time) and stride-to-stride variability parameters of gait (i.e., coefficient of variation for stride length or cycle time). The authors did not show any data on the variability of gait, so this sentence had better remove.

 2nd paragraph: Main focus of this paragraph was unknown. In the anterior part of this paragraph, the authors stated the gender difference in gait parameters, but in the posterior part of the paragraph, the difference between people who have a disorder and people who did not have a disorder. With the various topic included in one paragraph, it comes difficult to understand what the authors want to discuss here, and why the authors try to compare gender differences in spatiotemporal parameters of gait.

 3rd paragraph: Again, two topics are mixed. The former part is about gait and age, and the latter part is about gait and obesity. I would recommend the authors write a paragraph each topic separately.

**Method**

 Sample size: Regarding the sample size calculation, isn't the calculation shown here for the sample size calculation for the correlation analysis? Since this study mainly compared gait parameters between men and women, I wonder if the authors would be used the expected standard deviation of the gait parameters for the sample size calculation.

**Equipment**

 More explanation is needed about the measurement using the Physiology 4. What is the measurement frequency? Where and how is the sensor attached? What kind of gait parameters are obtained? etc.

**Procedure**

 I do not understand what "Foot speed" means. Please explain it in more detail. In addition, the gait parameters are usually expressed as the combined value of both sides of the legs, especially in gait speed and cadence. The authors had better show them.

**Statistical analysis**

 The authors are comparing groups by age and BMI, but the criteria for dividing into groups were unknown. Please clarify how the group was made.

**Results**

 Please delete the sentence like "Independent sample t-tests were conducted to compare...". It should be stated in the statistical analysis section of the methods.

 In tables 2 to 4 please show the number of samples by gender, age, or BMI group.

**Discussion**

 In the first paragraph, lines 1-2: I don't think it can be considered a major result because it is not a statistically significant result. The authors should discuss statistically significant results first. Also, in the second and third paragraphs, the authors state insignificant results first.

Is the work clearly and accurately presented and does it cite the current literature?

Partly

If applicable, is the statistical analysis and its interpretation appropriate?

Partly

Are all the source data underlying the results available to ensure full reproducibility?

Partly

Is the study design appropriate and is the work technically sound?

Partly

Are the conclusions drawn adequately supported by the results?

No

Are sufficient details of methods and analysis provided to allow replication by others?

Partly

Reviewer Expertise:

Physiotherapy

I confirm that I have read this submission and believe that I have an appropriate level of expertise to confirm that it is of an acceptable scientific standard, however I have significant reservations, as outlined above.

# Response

**Published:** 2022-12-05

**Authors:** Mohammad Ahsan

**Affiliations:** Imam Abdulrahman Bin Faisal University , Saudi Arabia

The following provides more specific comments by section:

**Abstract**

 Results: The authors stated, "Females were found with higher total double support and cadence than males." but this is not supported by the statistically significant result.

 Changed

 Conclusion: It's unclear what refers to "these findings". It refers to "no differences for gender”? or refers to "females showed higher cadence than males"? The authors should state the conclusion more clearly and it should be based on the statistical results.

 Changed

**Introduction**

 In the first paragraph, line 5: I think the authors should distinguish spatiotemporal parameters of gait (i.e., stride length or cycle time) and stride-to-stride variability parameters of gait (i.e., coefficient of variation for stride length or cycle time). The authors did not show any data on the variability of gait, so this sentence had better remove.

 Removed.

 2nd paragraph: Main focus of this paragraph was unknown. In the anterior part of this paragraph, the authors stated the gender difference in gait parameters, but in the posterior part of the paragraph, the difference between people who have a disorder and people who did not have a disorder. With the various topic included in one paragraph, it comes difficult to understand what the authors want to discuss here, and why the authors try to compare gender differences in spatiotemporal parameters of gait.

 Author wants to highlight the previous differences among gender for gait parameters.

 3rd paragraph: Again, two topics are mixed. The former part is about gait and age, and the latter part is about gait and obesity. I would recommend the authors write a paragraph each topic separately.

 Author separates both paragraphs, one for age effect on gait and another one is for body composition.

**Method**

 Sample size: Regarding the sample size calculation, isn't the calculation shown here for the sample size calculation for the correlation analysis? Since this study mainly compared gait parameters between men and women, I wonder if the authors would be used the expected standard deviation of the gait parameters for the sample size calculation.

 Sample size calculation removed

**Equipment**

 More explanation is needed about the measurement using the Physiology 4. What is the measurement frequency? Where and how is the sensor attached? What kind of gait parameters are obtained? etc.

 Information added

**Procedure**

 I do not understand what "Foot speed" means. Please explain it in more detail.

 Separate foot (left and right) speed.

 In addition, the gait parameters are usually expressed as the combined value of both sides of the legs, especially in gait speed and cadence.

 Yes, It's true; we measure all the mentioned parameters for both feet. All the outcome measures are according to the software outcome. For references:  https://www.youtube.com/watch?v=2jy7m6RxC9Q

 The authors had better show them.

**Statistical analysis**

 The authors compare groups by age and BMI, but the criteria for dividing into groups were unknown. Please clarify how the group was made.

 The age criteria were mentioned in table 3 as age range (18-25) and (30-38)

 The Body Mass Index (BMI) groups were classified according to the universal criteria **Underweight** (Less than 18.5), **Healthy (**18.5-24.9), **Overweight **(25-29.9), and **Obese** (Higher than 30).

**Results**

 Please delete the sentence like "Independent sample t-tests were conducted to compare...". It should be stated in the statistical analysis section of the methods.

 Deleted

 In tables 2 to 4 please show the number of samples by gender, age, or BMI group.

 Added

**Discussion**

 In the first paragraph, lines 1-2: I don't think it can be considered a major result because it is not statistically significant. The authors should discuss statistically significant results first. Also, in the second and third paragraphs, the authors state insignificant results first.

 Changed accordingly

# Peer review: Reviewer response for version 1

**DOI:** 10.5256/f1000research.54892.r82919

**Published:** 2021-08-17

**Authors:** Moazzam Hussain Khan

**Affiliations:** 1 Centre for Physiotherapy and Rehabilitation Sciences, Jamia Millia Islamia, New Delhi, Delhi, India

Please provide more information on the following:

1. How were the participants recruited for this study?
2. Not enough detail regarding Physiolog 4 - please expand on this.
3. Reliability and validity of the instruments used.
4. In the procedure: the Detecto scale should be replaced with a stadiometer cum weighing scale.
5. Provide full details for reference number 22.
6. What about flat feet - have you included this in the study?

Is the work clearly and accurately presented and does it cite the current literature?

Yes

If applicable, is the statistical analysis and its interpretation appropriate?

Yes

Are all the source data underlying the results available to ensure full reproducibility?

Yes

Is the study design appropriate and is the work technically sound?

Yes

Are the conclusions drawn adequately supported by the results?

Yes

Are sufficient details of methods and analysis provided to allow replication by others?

Yes

Reviewer Expertise:

Exercise physiology, Sports Injury, Chronobiology, biomechanics

I confirm that I have read this submission and believe that I have an appropriate level of expertise to confirm that it is of an acceptable scientific standard.

# Response

**Published:** 2022-12-05

**Authors:** Mohammad Ahsan

**Affiliations:** Imam Abdulrahman Bin Faisal University , Saudi Arabia

Please provide more information on the following:

- How were the participants recruited for this study?

Participants were recruited through an open advertisement within the university campus.

- Not enough detail regarding Physiolog 4 - please expand on this.

Physilog is a high-quality 3D accelerometer, 3D gyroscope and barometric pressure sensor. It provided raw 3D acceleration data at a sample rate of 128 Hz. It has an inbuilt SD memory card to store recorded data. Physilog application for gait was installed on a tablet/computer and connected with sensors. Stored data were transferred to the computer through the USB cable. 

- Reliability and validity of the instruments used.

Reference # 21,22.

- In the procedure: the Detecto scale should be replaced with a stadiometer cum weighing scale.

Replaced.

- Provide full details for reference number 22.

Provided.

- What about flat feet - have you included this in the study?

No, we did not determine flat feet in this study. Our focus was on the gait pattern.
